# Growth and Pubertal Status in Children with Type 1 Diabetes: A Cross-Sectional Comparison with Non-Diabetic Controls

**DOI:** 10.3390/diagnostics16203340

**Published:** 2026-10-14

**Authors:** Monica Simina Mihuta, Dana Stoian, Denisa Pescari, Lorena Alexandra Andris, Andreea Maria Pop, Jacqueline Alexandra Hurrelbrink, Marius Alexandru Chis, Oana-Alexandra Velea-Barta, Corina Paul

**Affiliations:** 1 IInd Pediatrics Clinic, “Victor Babeș” University of Medicine and Pharmacy, 300041 Timisoara, Romania; simina.mihuta@umft.ro (M.S.M.); paul.corina@umft.ro (C.P.); 2 Department of Pediatrics, “Pius Brinzeu” Emergency Clinical County Hospital, 300226 Timisoara, Romania; 3 Center for Molecular Research in Nephrology and Vascular Disease, “Victor Babeș” University of Medicine and Pharmacy, 300041 Timisoara, Romania; 4 Department of Advanced Ultrasound, DRD Medical Center, 300029 Timisoara, Romania; denisa.bostina@umft.ro; 5 IInd Department of Internal Medicine, “Victor Babeș” University of Medicine and Pharmacy, 300041 Timisoara, Romania; 6 Department of Diabetes, Nutrition and Metabolic Diseases, “Pius Brinzeu” Emergency Clinical County Hospital, 300226 Timisoara, Romania; 7 Department of Postgraduate Studies and Residency, “Victor Babeș” University of Medicine and Pharmacy, 300041 Timișoara, Romania; lorena.andris@rezident.umft.ro; 8 Medical School, “Victor Babeș” University of Medicine and Pharmacy, 300041 Timisoara, Romania; andreea.pop@student.umft.ro (A.M.P.); jacqueline.hurrelbrink@student.umft.ro (J.A.H.); 9 Pediatric Cardiology Department, Emergency Institute for Cardiovascular Diseases and Transplantation, 540136 Targu Mures, Romania; alexchis96@gmail.com; 10 Department of Odontotherapy and Endodontics, Faculty of Dental Medicine, “Victor Babeș” University of Medicine and Pharmacy, 300041 Timisoara, Romania; velea.oana@umft.ro

**Keywords:** continuous subcutaneous insulin infusion, glycated hemoglobin, insulin pump therapy, linear growth, metabolic control, multiple daily injections, pediatric diabetes, pubertal development, type 1 diabetes mellitus

## Abstract

**Background/Objectives:** Type 1 diabetes mellitus (T1DM) has historically been associated with impaired linear growth and delayed pubertal development due to chronic insulin deficiency. Advances in intensive insulin therapy and glucose monitoring may have substantially reduced these effects. This study evaluated growth and pubertal development in children with T1DM treated with multiple daily injections (MDIs) or continuous subcutaneous insulin infusion (CSII) compared with non-diabetic controls and investigated whether metabolic control was associated with anthropometric outcomes. **Methods:** This retrospective observational study included 177 children aged 3–18 years: 102 with T1DM (60 treated with MDI and 42 with CSII) and 75 non-diabetic hospital-based controls. Height SDS, BMI SDS, Tanner stage, biochemical parameters, and diabetes-related variables were analyzed. Comparisons, correlations and multiple linear regression analyses were performed to evaluate associations between metabolic control and anthropometric parameters. **Results:** No significant differences were observed between children with T1DM and healthy controls regarding height SDS, BMI SDS, or pubertal development. Likewise, no anthropometric differences were identified between the MDI and CSII groups. Fasting plasma glucose was significantly lower in the CSII group (*p* < 0.001), whereas HbA1c was comparable between treatment modalities. Neither HbA1c nor other routinely assessed metabolic parameters showed significant associations with height SDS or BMI SDS in correlation or multivariable regression analyses. **Conclusions:** Children and adolescents with T1DM showed no significant differences in height SDS, BMI SDS, or Tanner stage compared with controls, and similar anthropometric findings were observed in patients receiving MDI and CSII. These results suggest that major disturbances in attained height and pubertal status were not evident in this contemporary cohort of children with T1DM. However, given the retrospective cross-sectional design, longitudinal studies are needed to determine how contemporary diabetes management and long-term metabolic control influence growth trajectories and pubertal progression.

## 1. Introduction

Type 1 diabetes mellitus (T1DM) is one of the most common chronic diseases of childhood and adolescence, resulting from autoimmune destruction of pancreatic β-cells and subsequent absolute insulin deficiency [1,2,3]. Its incidence has increased steadily worldwide over the past two decades, with recent systematic reviews reporting a continued rise across Europe and other regions, making T1DM an increasingly important public health concern in pediatric populations [4,5,6].

Beyond its effects on glucose metabolism, insulin is a key anabolic hormone required for normal childhood growth and development. Together with growth hormone and insulin-like growth factor-1, insulin regulates longitudinal bone growth, protein synthesis, and normal pubertal maturation. In untreated or poorly controlled T1DM, portal insulin deficiency reduces hepatic IGF-1 production, alters the GH–IGF-1 axis, and may impair growth velocity and pubertal progression [7,8,9,10]. Historically, children with T1DM frequently exhibited delayed puberty, reduced adult height, and, in severe cases, growth failure associated with prolonged metabolic decompensation [10,11,12].

However, the management of pediatric T1DM has changed profoundly over recent decades. Intensive insulin therapy, rapid- and long-acting insulin analogs, continuous glucose monitoring, and continuous subcutaneous insulin infusion (CSII) have substantially improved glycemic control and reduced the burden of chronic hyperglycemia [13,14,15,16,17,18,19,20,21]. Current ISPAD Clinical Practice Consensus Guidelines emphasize that, when adequate metabolic control is achieved, most children and adolescents with T1DM are expected to attain normal linear growth and pubertal development [22]. Nevertheless, the available evidence remains heterogeneous. While older studies consistently reported impaired growth, more recent investigations suggest that these abnormalities have become considerably less frequent in the era of modern diabetes management, although concerns remain regarding the potential influence of persistent hyperglycemia, diabetes duration, and treatment modality on growth outcomes [16,17,22].

In addition, insulin pump therapy has been associated with improved glycemic control, increased time in range, and lower glycemic variability compared with multiple daily injections (MDIs), but whether these metabolic advantages translate into measurable differences in growth and pubertal development remains uncertain [14,17,18,19]. Data directly comparing anthropometric outcomes between contemporary MDI- and CSII-treated pediatric cohorts are still limited.

Therefore, the present study aimed to evaluate growth and pubertal development in children and adolescents with T1DM receiving either MDI or CSII compared with non-diabetic hospital-based controls and to investigate whether metabolic control and diabetes-related clinical characteristics are independently associated with anthropometric outcomes in a contemporary pediatric cohort. Although growth outcomes in children with T1DM have been investigated in previous studies, contemporary data integrating anthropometric outcomes, pubertal development, metabolic control, and insulin-delivery modality within the same pediatric cohort remain limited. The present study addresses these aspects simultaneously by comparing children with T1DM with a control group and by further evaluating patients receiving MDI and CSII, together with multivariable assessment of the relationship between metabolic and anthropometric parameters.

## 2. Materials and Methods

### 2.1. Study Design

This retrospective, observational, analytical study performed statistical analysis on a clinical database derived from routine medical practice. The study was conducted at the Department of Pediatrics of “Pius Brînzeu” Emergency Clinical County Hospital, Timișoara, Romania, by retrospectively reviewing the medical records of pediatric patients hospitalized between 2016 and 2025.

The local Research Ethics Committee of the “Pius Brînzeu” Emergency Clinical County Hospital, Timișoara has approved the study in accordance with the Helsinki Declaration (No. 600/12 February 2026).

### 2.2. Study Population and Inclusion Criteria

A total of 177 children aged 3–18 years were included and divided into three study groups:

• Control group: 75 non-diabetic hospital-based controls;
• MDI group: 60 children with T1DM receiving multiple daily insulin injections;

CSII group: 42 children with T1DM treated with continuous subcutaneous insulin infusion.

Inclusion Criteria:

Children aged between 3 and 18 years were eligible for inclusion.

Patients in the T1DM groups were required to have a confirmed diagnosis of type 1 diabetes for at least one year and to have been treated either with multiple daily insulin injections since diagnosis or with insulin pump therapy for at least six months. Complete data regarding anthropometric measurements and HbA1c were required for inclusion.

Exclusion criteria included:

• chronic diseases known to affect growth and development (e.g., endocrine or genetic disorders);
• diabetes types other than T1DM;
• incomplete medical records;
• chronic treatments potentially affecting growth (e.g., long-term corticosteroid therapy).

Control participants were selected retrospectively from children admitted to the same Department of Pediatrics during the study period for acute or minor conditions unrelated to chronic growth or pubertal disorders. Eligible controls had no known chronic disease or other underlying conditions considered likely to affect growth or pubertal development. Medical records and available laboratory investigations were reviewed, and participants with relevant abnormalities in routinely assessed inflammatory markers, liver enzymes, or renal function were excluded. Controls were selected to achieve an age and sex distribution broadly comparable to that of the T1DM cohort, without individual matching. Among children meeting these criteria, participants with complete anthropometric and laboratory data were included in the control group.

### 2.3. Data Collection

The following demographic and clinical variables were collected for all participants:

• age (years);
• sex;
• place of residence (urban/rural);
• body weight (kg), height (cm) and height standard deviation score (height SDS) (body weight and height were measured at hospital admission using the department’s standard calibrated weighing scales and stadiometers, with children undressed and without shoes);
• body mass index (BMI, kg/m^2^) and BMI standard deviation score (BMI SDS);
• pubertal development according to Tanner staging;
• blood parameters: fasting plasma glucose (mg/dL), serum creatinine (mg/dL), estimated glomerular filtration rate (eGFR, mL/min/1.73 m^2^, Schwartz method), aspartate aminotransferase (AST), alanine aminotransferase (ALT). Blood samples were collected a jeun, after 10–12 h overnight fasting as per department protocol, and analyzed in the hospital’s central laboratory using standard automated laboratory methods.

Pubertal development was assessed according to Tanner staging as documented in the clinical examination records. These assessments had been performed during routine clinical care by pediatricians working in the Department of Pediatrics and were retrospectively extracted from the medical records. As examinations were performed by multiple clinicians and no formal assessment of interobserver agreement was available, potential interobserver variability could not be quantified.

For children with T1DM, the following diabetes-related variables were additionally recorded:

• diabetes duration (years);
• insulin treatment modality (MDI or CSII);
• glycated hemoglobin (HbA1c %), total cholesterol, HDL cholesterol, LDL cholesterol, and triglycerides (mg/dL);
• diabetes-related comorbidities (overweight/obesity, hepatic steatosis, diabetic nephropathy, and insulin injection-site lipodystrophy) or associated autoimmune diseases (autoimmune thyroiditis, celiac disease).

For patients receiving insulin pump therapy, continuous glucose monitoring derived metrics were also analyzed, including:

• time in range (TIR, 70–180 mg/dL);
• coefficient of variation (CV);
• time spent in level 1 hypoglycemia—54–70 mg/dL;
• time spent in level 2 hypoglycemia—40–54 mg/dL;
• time spent in hyperglycemia—180–250 mg/dL;
• time spent in hyperglycemia—250–400 mg/dL;

Metabolic variables and diabetes-associated autoimmune diseases were evaluated exclusively in participants with T1DM and were analyzed descriptively rather than as primary study outcomes.

The CDC 2000 growth references were applied consistently to both study groups to allow standardized comparison of height and BMI SDS. Height SDS and BMI SDS were calculated using the LMS parameters derived from the Centers for Disease Control and Prevention 2000 growth charts, adjusted for age and sex [23].

### 2.4. Statistical Analysis

Data were entered into Microsoft Excel and analyzed using MedCalc Statistical Software version 20.111 (MedCalc Software Ltd., Ostend, Belgium) and Numiqo Online Statistics Calculator (e.U. Graz, Austria. URL https://numiqo.com).

Comparisons among the three study groups focused primarily on anthropometric parameters and pubertal development. Statistical analyses also evaluated the influence of insulin treatment modality and metabolic control on growth-related outcomes.

The distribution of continuous variables was assessed using the Shapiro–Wilk test. Normally distributed variables were analyzed using Student’s *t*-test (two-group comparisons) or one-way analysis of variance (ANOVA) (three-group comparisons), while Pearson’s correlation coefficient was used to assess associations between continuous variables. Non-normally distributed variables were analyzed using the Mann–Whitney *U* test (two-group comparisons) or the Kruskal–Wallis test (three-group comparisons), and correlations were evaluated using Spearman’s rank correlation coefficient. A two-sided *p* value < 0.05 was considered statistically significant.

Because pubertal status is an ordinal outcome strongly dependent on age and sex, additional ordinal logistic regression analyses were performed using pubertal category (Tanner I, Tanner II–IV, and Tanner V) as the dependent variable. The association between T1DM status and pubertal category was evaluated after adjustment for age and sex in the entire cohort. A separate analysis restricted to children with T1DM evaluated the association between insulin treatment modality (MDI vs. CSII) and pubertal category, also adjusting for age and sex.

Model assumptions were assessed by examining residual normality, homoscedasticity, multicollinearity (variance inflation factor, VIF), and influential observations before interpretation of the regression results.

No a priori sample size or power calculation was performed because of the retrospective study design; the sample size was determined by the number of eligible participants with complete data available during the predefined study period.

## 3. Results

### 3.1. Descriptive Analysis

A total of 177 children evaluated between 2016 and 2025 were included in the study. Of these, 102 children were diagnosed with T1DM, including 60 treated MDI and 42 receiving CSII. The control group consisted of 75 children without diabetes mellitus.

The distribution of all continuous variables was assessed using the Shapiro–Wilk test. Most variables showed a non-normal distribution (*p* < 0.05) and were therefore analyzed using non-parametric statistical methods. Variables that followed a normal distribution included height in all three study groups, fasting plasma glucose in the insulin pump group, HbA1c in the insulin pump group, and body weight in the insulin pump group. Accordingly, continuous data is presented as median (Q1–Q3) unless otherwise specified.

The sex distribution was 50–50% balanced. For place of residence, 51% of subjects come from urban areas. Assessment of pubertal development according to Tanner staging demonstrated representation across all three developmental categories, with the highest frequency observed in Tanner stages II–IV (Table 1).

Among children with T1DM, autoimmune thyroiditis was the most frequently associated autoimmune disorder, 21.5%, followed by overweight/obesity and insulin injection-site lipodystrophy in 12% of cases each. Celiac disease was only observed in 3 cases (3%), while diabetic kidney disease in only one diabetic patient (1%) (Table 1).

### 3.2. Comparison Analysis

The comparison between children with T1DM, regardless of insulin treatment modality, and controls revealed no statistically significant differences in anthropometric parameters or pubertal development. BMI SDS, height SDS, height, age, and Tanner stage distribution were comparable between the two groups.

As expected, fasting plasma glucose levels were significantly higher in children with T1DM than in controls (*p* < 0.001). Estimated GFR was also significantly higher in the T1DM group (*p* < 0.001); however, values remained within the normal range in both groups, indicating no clinically meaningful difference. No significant between-group differences were observed for serum creatinine, AST, or ALT. The results are summarized in Table 2.

Sex-stratified analyses showed no significant differences in height SDS between children with T1DM and controls. Among boys, mean height SDS was 0.57 ± 1.1 in the T1DM group (*n* = 51) and 0.55 ± 1.34 in the control group (*n* = 40, *p* = 0.93). Similarly, among girls, mean height SDS was 0.5 ± 0.98 in the T1DM group (*n* = 51) and 0.17 ± 1.21 in the control group (*n* = 35, *p* = 0.19).

In a multivariable linear regression model including T1DM status, sex, BMI SDS, and Tanner stage, T1DM status was not independently associated with height SDS (B = 0.156, 95% CI: −0.18 to 0.49, *p* = 0.36). BMI SDS was not significantly associated with height SDS (B = 0.11, 95% CI: −0.004 to 0.23, *p* = 0.058), and no significant associations were observed for sex or Tanner stage. The overall model was not statistically significant (R^2^ = 0.04, adjusted R^2^ = 0.02, F(4,172) = 1.97, *p* = 0.1.

Given the dependence of pubertal status on age and sex, an additional ordinal logistic regression analysis was performed using pubertal category (Tanner I, Tanner II–IV, and Tanner V) as the dependent variable. After adjustment for age and sex, T1DM status was not significantly associated with pubertal category (OR = 0.41, 95% CI: 0.11–1.58, *p* = 0.19).

Comparison of the three study groups (MDI, CSII, and healthy controls) revealed significant differences in age, fasting plasma glucose, serum creatinine, and eGFR. Fasting plasma glucose was highest in the MDI group (*p* < 0.001, Figure 1). Although the combined T1DM cohort was age-matched to the healthy controls (*p* = 0.34, Table 2), the CSII group was significantly older than the other groups (*p* = 0.04). Serum creatinine and eGFR also differed significantly among the three groups (*p* = 0.002 and *p* < 0.001, respectively), although both parameters remained within the normal range across all groups. In contrast, no significant differences were observed for BMI SDS, height, height SDS, Tanner stage, AST, or ALT. The results are summarized in Table 3.

Comparison between children treated with multiple daily injections and those receiving continuous subcutaneous insulin infusion demonstrated significant differences in fasting plasma glucose and serum creatinine levels. Children treated with MDI had significantly higher fasting plasma glucose concentrations than those receiving CSII (Figure 2), whereas serum creatinine values were significantly higher in the CSII group (both *p* < 0.001). No statistically significant differences were identified between treatment groups regarding height SDS, BMI SDS, HbA1c (Figure 3), eGFR, liver enzymes, total cholesterol, triglycerides, HDL cholesterol, or LDL cholesterol.

Because the CSII group was significantly older than the MDI group, pubertal status was additionally evaluated using ordinal logistic regression adjusted for age and sex. In this analysis, CSII treatment was associated with lower odds of being in a more advanced pubertal category compared with MDI (OR = 0.07, 95% CI: 0.006–0.89, *p* = 0.04). However, the estimate was imprecise, as reflected by the wide confidence interval, and should be interpreted cautiously given the non-randomized treatment allocation and previous MDI exposure of all patients currently receiving CSII.

The results are presented in Table 4.

**Figure 2:**
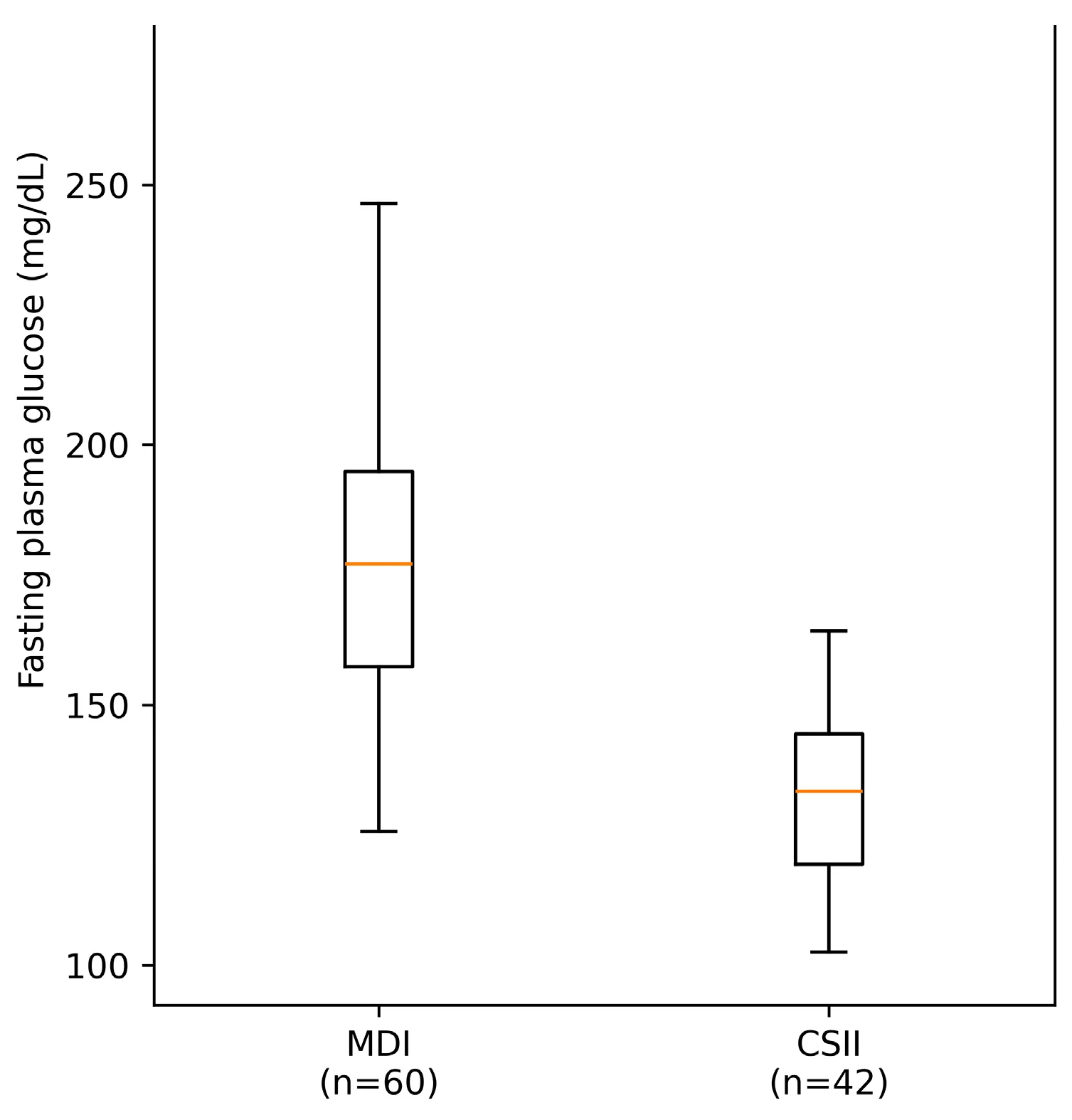
Fasting glucose (mg/dL) levels in the MDI vs. CSII groups (*p* < 0.001).

**Figure 3:**
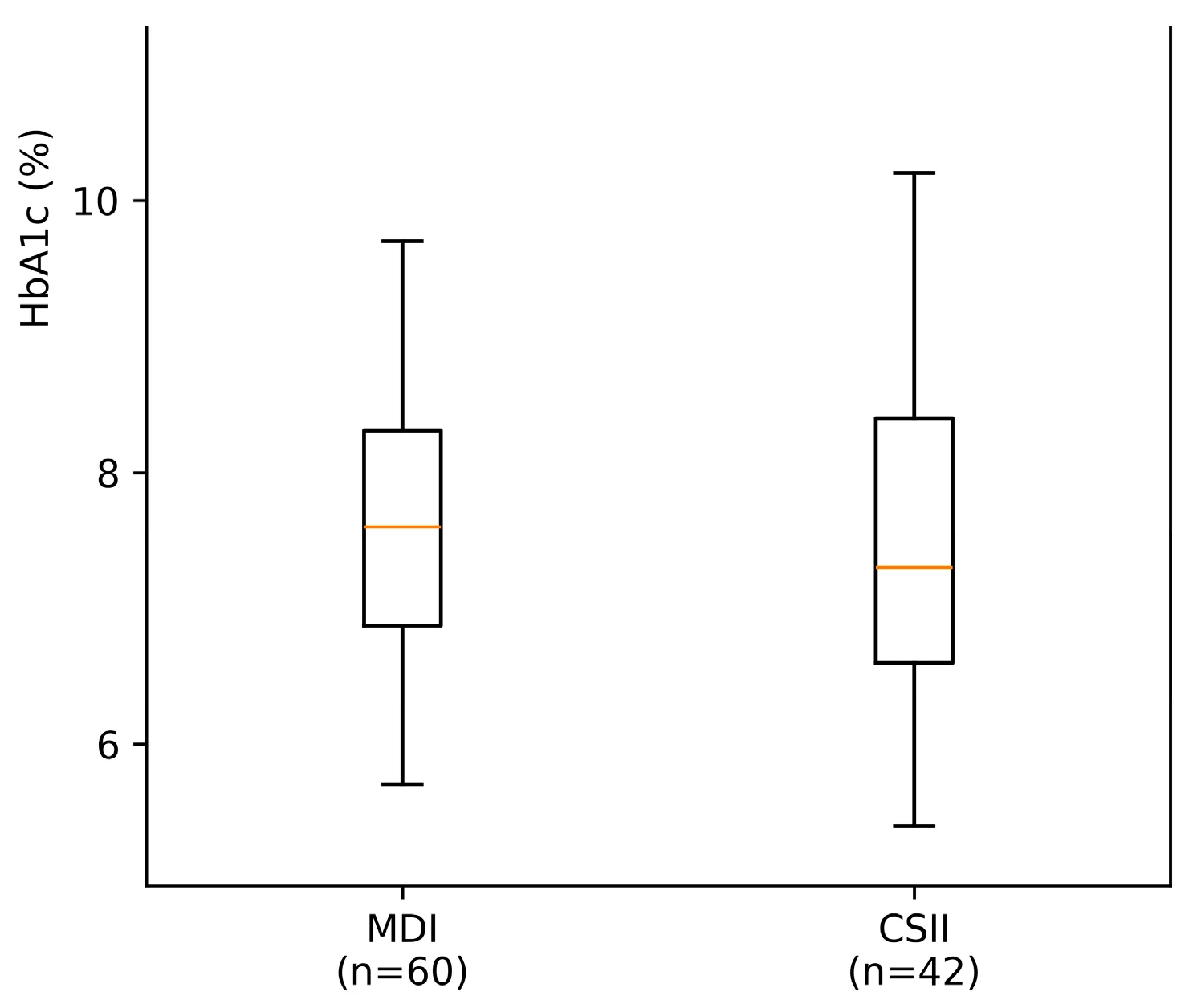
HbA1c (%) levels in the MDI vs. CSII groups (*p* = 0.4).

Continuous glucose monitoring parameters (CGM) were compared among prepubertal (Tanner I), pubertal (Tanner II–IV), and post-pubertal (Tanner V) children treated with continuous subcutaneous insulin infusion using the Kruskal–Wallis test. A statistically significant difference was observed only for the percentage of time spent in hyperglycemia within the 180–250 mg/dL range (*p* = 0.03). Prepubertal children exhibited a higher proportion of time in this glycemic range than pubertal and post-pubertal participants. Although the percentage of TIR tended to increase with pubertal stage, the difference did not reach statistical significance (*p* = 0.05). Likewise, no statistically significant differences were identified among Tanner stages regarding HbA1c, time spent in hyperglycemia, time spent in hypoglycemia, BMI SDS, or height SDS. The results are presented in Table 5.

### 3.3. Correlation Analysis

Spearman correlation analysis revealed no statistically significant associations between BMI SDS and age, Tanner stage, fasting plasma glucose, serum creatinine, eGFR, AST, or ALT.

Height SDS showed a weak positive correlation with eGFR (ρ = 0.19, *p* = 0.01). No significant correlations were observed between height SDS and age, Tanner stage, fasting plasma glucose, serum creatinine, AST, or ALT. The results are presented in Table 6.

Spearman correlation analysis performed in the combined T1DM cohort (MDI and CSII groups) demonstrated no statistically significant associations between BMI SDS and age, duration of diabetes, Tanner stage, HbA1c, fasting plasma glucose, serum creatinine, eGFR, liver enzymes, or lipid profile.

Similarly, height SDS was not significantly correlated with any of the evaluated clinical or biochemical parameters. The strongest association was observed between height SDS and eGFR (ρ = 0.19, *p* = 0.05), although this did not reach statistical significance. Likewise, duration of diabetes showed a weak inverse correlation with height SDS (ρ = −0.181, *p* = 0.07), but the association was not statistically significant. All remaining correlations were weak and non-significant (Table 7).

Spearman correlation analysis performed in children receiving CSII demonstrated a single statistically significant association. A weak positive correlation was observed between the percentage of time spent in hypoglycemia (54–70 mg/dL) and height SDS (ρ = 0.34, *p* = 0.02), indicating that children with a higher height SDS tended to spend a greater proportion of time within this hypoglycemic range, a result which is not necessarily in line with what was expected. No significant correlations were identified between height SDS and HbA1c, Time in Range (TIR), time spent in hypoglycemia below 54 mg/dL, time spent in hyperglycemia (180–250 mg/dL or >250 mg/dL), or coefficient of variation. Similarly, BMI SDS was not significantly associated with any of the evaluated continuous glucose monitoring parameters or HbA1c. The results are presented in Table 8.

### 3.4. Regression Analysis

To identify factors independently associated with BMI SDS, a multiple linear regression analysis was performed using BMI SDS as the dependent variable. Age, sex, place of residence, Tanner stage, fasting plasma glucose, eGFR, AST, and ALT were included as independent variables. The analysis included all 177 participants. The overall regression model was not statistically significant (R^2^ = 0.05, adjusted R^2^ = 0.009, F(8,168) = 1.21, *p* = 0.29), indicating that the selected variables explained only a small proportion of the variability in BMI SDS. Among the individual predictors, only place of residence was significantly associated with BMI SDS (*p =* 0.01), whereas age, sex, Tanner stage, fasting plasma glucose, eGFR, AST, and ALT were not independently associated with BMI SDS. Assessment of multicollinearity demonstrated acceptable variance inflation factors, ranging from 1.03 to 6.07, indicating no evidence of problematic multicollinearity among the independent variables (Table 9).

To identify factors independently associated with height SDS, a multiple linear regression analysis was performed using height SDS as the dependent variable. Age, sex, place of residence, BMI SDS, Tanner stage, fasting plasma glucose, eGFR, AST, and ALT were included as independent variables. The analysis included all 177 participants. The overall regression model was not statistically significant (R^2^ = 0.07, adjusted R^2^ = 0.02, F(9,167) = 1.58, *p* = 0.12), indicating that the included variables explained only a small proportion of the variability in height SDS. Among the individual predictors, BMI SDS (*p* = 0.01) and place of residence (*p* = 0.04) were independently associated with height SDS, whereas age, sex, Tanner stage, fasting plasma glucose, eGFR, AST, and ALT were not significantly associated with height SDS. VIF ranged from 1.06 to 6.07, indicating no evidence of problematic multicollinearity among the independent variables (Table 10).

A multiple linear regression analysis was performed to evaluate the independent associations of metabolic control variables with height SDS in children and adolescents with T1DM. The final model included fasting plasma glucose, HbA1c, eGFR, ALT, LDL-c, HDL-c, triglycerides, and treatment modality (CSII versus MDI) as independent predictors of height SDS. To reduce multicollinearity and improve model stability, total cholesterol and AST were excluded from the final multiple linear regression model. However, the overall regression model was not statistically significant (R^2^ = 0.05, adjusted R^2^ = −0.02, F(8,93) = 0.66, *p* = 0.72), indicating that these variables explained only a small proportion of the variability in height SDS. None of the evaluated predictors independently predicted height SDS after adjustment for the remaining covariates (Table 11).

In a multivariable linear regression model including T1DM status, sex, BMI SDS, and Tanner stage, T1DM status was not independently associated with height SDS (B = 0.156, 95% CI: −0.185 to 0.49, *p* = 0.36). BMI SDS was also not significantly associated with height SDS (B = 0.11, 95% CI: −0.004 to 0.23, *p* = 0.058), and no significant associations were observed for sex or Tanner stage. The overall model was not statistically significant (R^2^ = 0.04, adjusted R^2^ = 0.02, F(4,172) = 1.97, *p* = 0.1.

To evaluate whether glycemic control, assessed by HbA1c, represents an independent predictor of linear growth in children and adolescents with T1DM, a multiple linear regression model was constructed with height SDS as the dependent variable. The model was adjusted for age, sex, diabetes duration, and insulin therapy modality (CSII vs MDI). The overall regression model was not statistically significant (R^2^ = 0.05, adjusted R^2^ = −0.001, F(5,96) = 0.99, *p* = 0.42), indicating that the included variables explained only a small proportion of the variability in height SDS. HbA1c was not independently associated with height SDS after adjustment for potential confounding factors. Likewise, age, sex, diabetes duration, and insulin therapy modality were not significant independent predictors of height SDS, although diabetes duration showed a non-significant trend toward a negative association with height SDS (*p* = 0.08). The results of the regression analysis are presented in Table 12.

Among the comorbidities of the T1DM patients, autoimmune thyroiditis was considered the most clinically relevant potential confounder of linear growth with a sufficient number of affected participants to permit inclusion in a sensitivity analysis. Celiac disease, hepatic steatosis, and diabetic nephropathy were not included as separate covariates because of the small number of affected participants. Lipodystrophy was not considered a direct systemic growth-related confounder, while the potential influence of overweight/obesity was accounted for by including BMI SDS as a continuous variable in the regression model rather than the binary overweight/obesity classification. See Table 1.

Among the 102 children with T1DM, autoimmune thyroiditis was present in 23. Mean height SDS did not differ significantly between children with and without autoimmune thyroiditis (0.68 ± 1 vs. 0.49 ± 1.05, respectively, *p* = 0.36, Mann–Whitney test).

To further evaluate autoimmune thyroiditis as a potential confounder of growth, a multivariable linear regression analysis was performed within the T1DM cohort, with height SDS as the dependent variable and autoimmune thyroiditis, sex, BMI SDS, Tanner stage, and treatment modality (MDI vs. CSII) as independent variables. After adjustment, autoimmune thyroiditis was not significantly associated with height SDS (B = 0.27, 95% CI: −0.25 to 0.79, *p* = 0.3). Treatment modality was also not significantly associated with height SDS (B = 0.17, 95% CI: −0.26 to 0.61, *p* = 0.42), and no significant associations were observed for sex, BMI SDS, or Tanner stage. The overall regression model was not statistically significant (R^2^ = 0.05, adjusted R^2^ = −0.032, F(8,93) = 0.61, *p* = 0.76).

As an additional sensitivity analysis, all 23 T1DM patients with autoimmune thyroiditis were excluded, leaving 79 children with T1DM and 75 controls. Mean height SDS remained comparable between T1DM patients without autoimmune thyroiditis and controls (0.49 ± 1.05 vs. 0.37 ± 1.29, respectively, *p* = 0.53). The multivariable linear regression analysis was subsequently repeated in this restricted cohort, with height SDS as the dependent variable and T1DM status, sex, BMI SDS, and Tanner stage as independent variables. T1DM status remained unrelated to height SDS after exclusion of patients with autoimmune thyroiditis (B = 0.009, 95% CI: −0.4 to 0.42, *p* = 0.96). BMI SDS was positively associated with height SDS (B = 0.156, 95% CI: 0.03 to 0.28, *p* = 0.01), whereas no significant associations were observed for sex or Tanner stage. The overall model did not reach statistical significance (R^2^ = 0.08, adjusted R^2^ = 0.04, F(7,146) = 1.92, *p* = 0.07). These findings indicate that exclusion of T1DM patients with autoimmune thyroiditis did not alter the observed lack of association between T1DM status and height SDS.

## 4. Discussion

Type 1 diabetes mellitus is one of the most common chronic metabolic diseases of childhood and adolescence and represents a major challenge for patients, their families, and healthcare systems. This condition is characterized by the autoimmune destruction of pancreatic β-cells, resulting in absolute insulin deficiency and disturbances in carbohydrate, lipid, and protein metabolism [1].

Insulin plays an essential role in anabolic processes, being involved in cellular glucose utilization, stimulation of protein synthesis, and the maintenance of normal growth and development [7]. It acts directly on peripheral tissues and indirectly through the IGF-1 pathway, which mediates many of the growth-promoting effects of GH. In T1DM, reduced portal insulin availability may decrease hepatic GH responsiveness and IGF-1 production, resulting in a state of relative GH resistance characterized by increased GH secretion but reduced circulating IGF-1 concentrations. Alterations in IGF-binding proteins may further reduce IGF-1 bioavailability. These abnormalities are more pronounced in patients with poor metabolic control and may contribute to impaired linear growth, particularly during puberty, when activation of the GH–IGF-1 axis is physiologically enhanced. Adequate insulin replacement and improved metabolic control can partially restore this axis, helping to preserve normal linear growth of the findings [24,25].

The present study aimed to evaluate growth and pubertal status in children and adolescents with T1DM and to investigate their relationship with metabolic control. In this contemporary cohort, no statistically significant differences were observed between children with T1DM and controls regarding height SDS, BMI SDS, or Tanner stage. Furthermore, no independent associations were identified between routinely assessed markers of metabolic control and the anthropometric parameters evaluated. These findings suggest that major disturbances in attained height and pubertal status were not evident in the children with T1DM included in our cohort, although the cross-sectional design does not allow assessment of individual growth velocity or pubertal progression over time. Although these findings may initially appear inconsistent with classical literature [26,27], they agree with the evolution of T1DM management over the last two decades. Studies performed during the 1980s and 1990s frequently reported reduced final height and delayed pubertal development in children with T1DM. However, these cohorts consisted mainly of patients treated before the introduction of intensive insulin therapy and modern glucose monitoring technologies [26,27]. More recent evidence provides a more nuanced picture of growth in children with T1DM [22,28]. Longitudinal studies conducted in the era of intensive insulin therapy have shown that final height may remain close to genetic target height despite subtle changes in height SDS during childhood and puberty. Nevertheless, residual growth alterations have not been eliminated, and recent pooled evidence suggests a small reduction in final height SDS among individuals with T1DM. Similarly, contemporary registry data indicate that pubertal timing in children with T1DM is influenced by several factors, including metabolic control, BMI, and diabetes duration. Taken together, these findings suggest that growth and pubertal outcomes in contemporary T1DM populations are generally more favorable than historically reported, while remaining heterogeneous and potentially influenced by both disease-related and individual factors. The absence of significant differences in height SDS, BMI SDS, and Tanner stage in our cohort is consistent with this evolving literature, but the cross-sectional design of the present study does not allow conclusions regarding individual growth trajectories or the causal contribution of contemporary diabetes management [29,30]. Hence, an important aspect of the present study is that all children included in the T1DM cohort were diagnosed and treated between 2016 and 2025, a period during which the management of pediatric T1DM was based predominantly on intensive insulin therapy, modern insulin analogs, continuous glucose monitoring, and, in a substantial proportion of patients, insulin pump therapy. Within this clinical context, it is plausible that exposure to severe and prolonged hyperglycemia was insufficient to produce measurable alterations in growth and development, thereby explaining the absence of significant differences between children with T1DM and healthy controls. Our findings are further supported by the current ISPAD Clinical Practice Consensus Guidelines, which emphasize that most children and adolescents with T1DM treated with contemporary intensive insulin regimens, including multiple daily injections or continuous subcutaneous infusion, achieve normal linear growth [16]. According to these recommendations, reduced growth velocity is observed mainly in patients with persistent poor metabolic control, in whom prolonged insulin deficiency disrupts the growth hormone–IGF-1 axis and decreases circulating IGF-1 concentrations. Furthermore, the adverse effects of chronic hyperglycemia on growth become more evident during puberty and in patients who develop albuminuria or Mauriac syndrome [16].

The potential influence of insulin delivery modality on growth remains incompletely defined. Large observational data from the SWEET registry suggested slightly higher adjusted height z-scores among insulin pump users, although glycemic control, diabetes duration, and age were also independently associated with growth outcomes [31]. Other longitudinal studies have shown largely preserved growth and pubertal development with contemporary intensive insulin therapy, whether delivered by MDI or CSII [32]. In contrast, the randomized SCIPI trial found no significant differences in growth outcomes between CSII and MDI during 12 months of follow-up [33]. Therefore, current evidence does not establish a consistent independent effect of insulin delivery modality on linear growth. In our cohort, height SDS, BMI SDS, and pubertal stage were likewise comparable between the MDI and CSII groups. One of the most important findings of the present study was the absence of an independent association between markers of metabolic control and anthropometric parameters. Both the correlation analyses and the multiple linear regression models failed to demonstrate significant associations between HbA1c or other routinely assessed metabolic parameters and either height SDS or BMI SDS. These findings should not be interpreted as evidence that glycemic control has no influence on growth. Rather, they suggest that the level of metabolic control achieved in our cohort was sufficient to prevent the growth impairment described in historical studies. In other words, the degree and duration of metabolic imbalance in our patients were likely insufficient to produce measurable alterations in growth and development.

Comparison between patients treated with multiple daily injections and those receiving insulin pump therapy revealed differences in some metabolic parameters but not in anthropometric outcomes. HbA1c values were comparable between the two treatment groups, suggesting that both therapeutic strategies allowed similar overall metabolic control in routine clinical practice. Although fasting plasma glucose was lower in patients treated with insulin pump therapy, this finding was not accompanied by differences in height SDS, BMI SDS, or pubertal development. These results should be interpreted with caution because the two treatment groups were not fully comparable with respect to age and pubertal status, factors that may independently influence several of the metabolic and biological parameters evaluated. Furthermore, for the CSII group, being significantly older than the MDI group, this fact has influenced pubertal stage, and may have influenced biochemical parameters, and anthropometric measures independently of treatment modality. Consequently, differences observed between MDI and CSII groups should be interpreted primarily as cross-sectional associations and not as effects attributable to the insulin-delivery modality itself.

Current evidence indicates that insulin pump therapy is generally associated with improved glycemic control, increased time in range, lower HbA1c levels, and a reduced risk of severe hypoglycemia compared with multiple daily injections. However, its benefits with respect to linear growth are considerably less evident. Most studies have shown that both continuous subcutaneous insulin infusion and basal-bolus therapy support normal growth and development when accompanied by adequate metabolic control [19,34,35]. The findings of our study are consistent with these observations. The absence of anthropometric differences between the two treatment groups suggests that, in contemporary clinical practice, both multiple daily injections and insulin pump therapy can provide the conditions necessary for normal physical development when treatment is appropriately implemented and accompanied by regular metabolic monitoring.

In the present study, no significant differences in BMI SDS were observed between children with T1DM and healthy controls. This finding is particularly relevant because recent studies have reported an increasing prevalence of obesity among children and adolescents with T1DM, a phenomenon attributed both to intensive insulin therapy and to behavioral and lifestyle factors [36,37]. The fact that this trend was not evident in our cohort may suggest an appropriate balance between insulin therapy, nutritional follow-up, and diabetes education. Furthermore, the absence of significant associations between routinely assessed metabolic parameters and BMI SDS in our multivariable analyses indicates that body weight in children with T1DM is likely influenced by a broader range of factors than glycemic control alone, including dietary habits, physical activity, genetic predisposition, and overall lifestyle.

In our study, pubertal development assessed according to Tanner staging did not differ significantly between children with T1DM and healthy controls. This finding supports the conclusion that, within our cohort, T1DM was not associated with measurable delays in pubertal maturation. Earlier studies frequently reported delayed puberty and reduced adult height in children with T1DM, particularly in patients managed with conventional insulin regimens and suboptimal metabolic control. For example, Elamin and colleagues described impaired linear growth and delayed sexual maturation in children receiving conventional insulin therapy [27]. In contrast, more recent evidence indicates that pubertal development is generally preserved in children with T1DM when intensive insulin therapy and satisfactory metabolic control are achieved. Although T1DM may influence the growth hormone–IGF-1 axis and pubertal progression, contemporary studies consistently demonstrate that most children treated with modern intensive therapeutic strategies achieve normal adult height and timely puberty [25]. Our findings agree with this evidence, as the absence of differences in Tanner stage between the study groups suggests that the standard of diabetes care provided was sufficient to prevent clinically relevant delays in pubertal development.

The multiple linear regression analyses confirmed the results of the univariate analyses, as no independent clinical or metabolic predictors of anthropometric parameters were identified. In all regression models, the low coefficients of determination indicate that only a small proportion of the variability in height SDS and BMI SDS could be explained by the clinical and metabolic variables included in the analyses. Neither HbA1c nor the other routinely assessed metabolic parameters independently predicted height SDS after adjustment for the selected covariates. These findings are consistent with the current understanding that growth and development are multifactorial processes influenced by the complex interaction of genetic, endocrine, nutritional, environmental, and metabolic factors. Therefore, under conditions of appropriate T1DM management, routinely measured metabolic markers such as HbA1c, fasting plasma glucose, lipid profile, renal function, or liver enzymes should not be considered the sole determinants of linear growth or nutritional status [16,22].

The prevalence of autoimmune thyroiditis in our cohort (21.6%) was comparable to that reported in the literature, where it represents the most common autoimmune disorder associated with childhood T1DM. As most patients were diagnosed and monitored according to current recommendations, early detection and appropriate thyroid hormone replacement therapy in those who developed hypothyroidism likely minimized the impact of this comorbidity on growth and development, which may explain the absence of anthropometric differences observed in our study [38,39]. Celiac disease was identified in 2.9% of children with T1DM, a lower prevalence than that reported in international pediatric registries, which goes up to over 10%, depending on ethnicities and geographic regions [38,40,41]. Although untreated celiac disease may impair growth velocity and nutritional status, the small number of affected patients and the timely initiation of a gluten-free diet did not allow the identification of a measurable impact on anthropometric parameters within our cohort [38]. The proportion of children with overweight or obesity (12.7%) was lower than that reported in many contemporary pediatric cohorts, where the prevalence of excess body weight in children with T1DM frequently exceeds 20% [36,42]. This finding may reflect the characteristics of the study population as well as the regular nutritional counseling and follow-up provided at our center. Diabetic kidney disease was identified in only one patient, a frequency too low to permit evaluation of its potential influence on growth and development [43]. Injection-site lipodystrophy was observed in approximately 13% of patients, a prevalence lower than that reported in many pediatric studies [44]. This finding may reflect the effectiveness of diabetes education regarding injection-site rotation and the regular clinical follow-up performed at our institution.

From a clinical perspective, the findings of the present study provide an encouraging message for both healthcare professionals and families of children diagnosed with T1DM. Whereas in the past this diagnosis was frequently associated with concerns regarding impaired linear growth and delayed pubertal development, our data suggest that these complications can largely be prevented through contemporary diabetes management. Intensive insulin therapy, careful metabolic monitoring, structured diabetes education, and regular follow-up in a specialized pediatric diabetes center allow most children and adolescents with T1DM to achieve growth and development comparable to that of their healthy peers. These findings have important implications for patient and family counseling from the time of diagnosis and reinforce the importance of maintaining high standards of diabetes care throughout childhood and adolescence.

### Strengths and Limitations

The main strength of the present study is the inclusion of a cohort of children managed exclusively during the era of intensive insulin therapy and modern glucose monitoring technologies, allowing the evaluation of the current impact of T1DM on growth and development. Furthermore, the study assessed not only anthropometric outcomes but also their relationship with a broad range of routinely evaluated metabolic parameters using multivariable regression analyses. This approach enabled the investigation of the independent contribution of metabolic control while accounting for the potential influence of other clinical variables.

Nevertheless, several limitations should be acknowledged. The retrospective design precludes the establishment of causal relationships between metabolic control and anthropometric outcomes. The lack of retrospective data on pubertal onset, growth velocity, bone age, and target height represents serious limitations of the analysis. The Tanner stage evaluation performed by multiple pediatricians could also represent potential interobserver variability that could not be quantified. Pubertal assessment in the present study was limited to Tanner stage documented at the clinical evaluation included in the analysis and should be interpreted as a cross-sectional assessment of pubertal status rather than an evaluation of pubertal progression.

The study was conducted at a single tertiary pediatric center, which may limit the generalizability of the findings.

In addition, the MDI and CSII groups were not fully comparable regarding age and pubertal development, factors that may have influenced some of the observed metabolic and biological differences. Treatment allocation was not randomized, and all patients in the CSII group had previously received MDI, with a median CSII treatment duration of 3.0 years (IQR 2.0–5.0 years). Therefore, the absence of statistically significant differences in height SDS and BMI SDS between the current MDI and CSII groups should not be interpreted as evidence of equivalent effects of the two insulin-delivery modalities on growth. Because of the cross-sectional design and the absence of detailed longitudinal growth and metabolic data before and after CSII initiation, the study cannot determine whether insulin-delivery modality itself influences growth trajectories.

Moreover, metabolic control was evaluated primarily using HbA1c together with routinely available laboratory parameters collected at the time of assessment, without quantifying cumulative long-term exposure to hyperglycemia or incorporating continuous glucose monitoring metrics such as time in range. Glycemic control was assessed using HbA1c values available at the study evaluation, while longitudinal HbA1c measurements were not systematically available. Therefore, mean HbA1c over the course of diabetes or other measures of cumulative glycemic exposure could not be evaluated. Similarly, CGM-derived metrics, including time in range and glycemic variability, reflected the period available at study evaluation and were available only for the CSII group. Consequently, the present cross-sectional metabolic measures may not fully represent long-term glycemic control during the period relevant to linear growth and pubertal development.

The use of CDC 2000 growth references rather than population-specific Romanian or contemporary European growth charts represents an additional limitation, as differences between reference populations may influence calculated anthropometric SDS values. Nevertheless, CDC growth references are widely recognized and have been used in European pediatric research, and their consistent application to both the T1DM and control groups allows standardized between-group comparisons. Therefore, while the choice of reference may affect absolute SDS values, it is less likely to substantially influence the internal comparison between the study groups.

Finally, information regarding socioeconomic status, dietary habits, physical activity, birth weight, and parental height was not systematically recorded for either children with T1DM or control participants and could therefore not be incorporated into the analysis. As these factors may influence nutritional status, linear growth, and pubertal status, residual confounding related to these unmeasured variables cannot be excluded. In particular, the absence of parental height prevented calculation of target height and assessment of attained height in relation to each child’s genetic growth potential. Consequently, the absence of a statistically significant difference in height SDS between the study groups should be interpreted at the population level and does not establish that individual children with T1DM achieved their genetically expected height.

## 5. Conclusions

The present study evaluated growth and pubertal status in children and adolescents with T1DM receiving contemporary intensive insulin therapy and examined their relationship with metabolic control and insulin-delivery modality. In this cohort, no significant differences were observed between children with T1DM and controls regarding height SDS, BMI SDS, or Tanner stage. Similarly, height SDS and BMI SDS did not differ significantly between patients currently treated with MDI and those receiving CSII. Neither HbA1c nor the other routinely assessed metabolic parameters were independently associated with anthropometric outcomes.

These findings indicate that major disturbances in attained height and pubertal status were not evident in children and adolescents with T1DM. However, the cross-sectional design does not allow assessment of individual growth trajectories or pubertal progression, and the absence of parental height precluded evaluation of attained height in relation to genetic growth potential. Furthermore, because treatment allocation was not randomized, and patients receiving CSII were older and had previously been treated with MDI, the absence of significant anthropometric differences between the MDI and CSII groups should not be interpreted as evidence of equivalent effects of the two treatment modalities on growth.

Overall, our findings provide a contemporary cross-sectional perspective on growth and pubertal status in pediatric T1DM and are consistent with the increasingly favorable growth outcomes reported in the era of intensive diabetes management. Longitudinal studies incorporating growth velocity, pubertal progression, genetic target height, and long-term measures of glycemic exposure are needed to further define the relationships between metabolic control, insulin-delivery modality, and growth throughout childhood and adolescence.

## Figures and Tables

**Figure 1:**
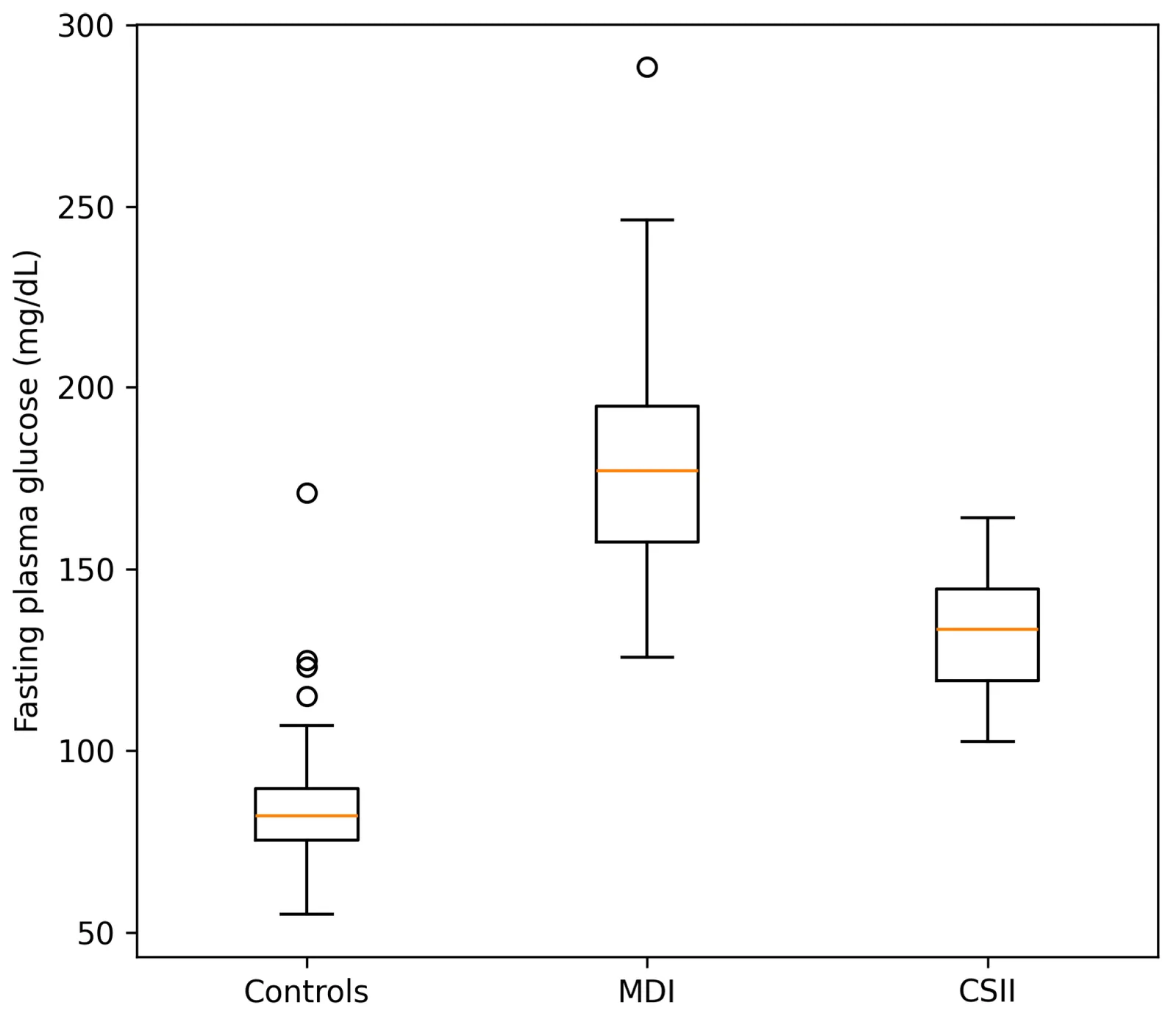
Fasting glucose (mg/dL) levels across the three study groups (*p* < 0.001).

**Table 1:** Demographic and clinical characteristics of the study population.

Parameter		Total (*n*)	MDI		CSII		Controls	
			*n*	%	*n*	%	*n*	%
Sex	Feminine	86	26	41.67	25	59.52	35	46.66
	Masculine	91	34	58.33	17	40.47	40	53.33
Place of residence	Urban	93	27	45	27	64.28	39	52
	Rural	84	33	55	15	35.71	36	48
Tanner Stage	Tanner I	48	15	25	8	19.04	25	33.33
	Tanner II–IV	67	31	51.66	15	35.71	21	28
	Tanner V	62	14	23.33	19	45.23	29	38.66
Autoimmune thyroiditis	Present	23	14	23.33	8	19.04	-	-
Overweight/obesity	Present	13	7	11.66	6	14.28	-	-
Celiac disease	Present	3	0	0	3	7.14	-	-
Diabetic kidney disease	Present	1	1	1.66	0	0	-	-
Insulin injection-site lipodystrophy	Present	13	8	13.33	5	11.9	-	-

**Table 2:** Comparison of clinical and usual blood tests in T1DM group vs. controls.

	T1DM (*n* = 102)	Controls (*n* = 75)	Mann–Whitney-U Test	
Parameter	Medians (Q1-Q3)		U	*p*-Value
Age, years	13.1 (10.5–15.0)	12.0 (8.54–15.0)	4145.5	0.34
Tanner stage	3 (2–5)	3 (1–5)	3812.0	0.96
BMI SDS	0.71 (−0.08–1.21)	0.53 (−0.61–1.40)	3999	0.6
Height, cm	160 (143.25–168.5)	155 (135–167)	4145.5	0.34
Height SDS	0.69 (−0.19–1.15)	0.35 (−0.61–1.42)	4136	0.36
Fasting glucose, mg/dL	152(134.95–182.85)	82 (75.5–89.5)	7535.5	<0.001
Creatinine, mg/dL	0.6 (0.5–0.71)	0.56 (0.49–0.7)	4111	0.39
eGFR, ml/min/1.73	118 (102.23–140)	104 (91.03–113.68)	5407	<0.001
AST, U/L	21 (18–27.75)	24 (19–31)	3396.5	0.2
ALT, U/L	19 (16–22.75)	17 (13–24.50)	4179	0.29

Mann–Whitney-*U* test; T1DM, type 1 diabetes melitus; BMI, body mass index; SDS, standard deviation, eGFR, estimated glomerular filtration rate (Scwartz method); ALT, alanine aminotransferase; AST, aspartate aminotransferase.

**Table 3:** Comparison of clinical and usual blood tests in the three study groups.

	MDI	CSII	Controls	Kruskal–Wallis Test	
Parameter	Medians (Q1–Q3)			H	*p*-Value
Age, years	12.85 (9.63–14.05)	14.35 (11.7–15.95)	12 (8.54–15)	6.48	0.04
Tanner stage	2 (2–4)	4 (2–5)	3 (1–5)	3.69	0.15
BMI SDS	0.65 (−0.34–1.19)	0.77 (0.22–1.21)	0.53 (−0.61–1.4)	0.87	0.64
Height, cm	157.45 (139.12–165.73)	162.9 (149–169.38)	155 (135–167)	3.89	0.14
Height SDS	0.65 (−0.39–1.17)	0.7 (−0.03–1.14)	0.35 (−0.61–1.42)	1.009	0.6
Fasting glucose, mg/dL	177.1 (157.38–194.85)	133.45 (119.32–144.45)	82 (75.50–89.5)	140.09	<0.001
Creatinine (mg/dL)	0.56 (0.46–0.6)	0.7 (0.57–0.74)	0.57 (0.49–0.7)	12.36	0.002
eGFR (ml/min/1.73)	118 (105.5–140)	115.5 (98.16–141.25)	104.56 (91.03–113.68)	22.31	<0.001
AST (U/L)	21 (18–28.25)	21.5 (17–25.75)	22 (19–31)	1.65	0.43
ALT (U/L)	19 (16–23.25)	18 (15.25–20.75)	17 (13–24.5)	1.65	0.43

Kruskal–Wallis test; MDI, multiple daily injections; CSII, continuous subcutaneous insulin infusion; BMI, body mass index; SDS, standard deviation, eGFR, estimated glomerular filtration rate (Scwartz method); ALT, alanine aminotransferase; AST, aspartate aminotransferase.

**Table 4:** Comparison of clinical and usual blood tests in MDI vs. CSII groups.

Parameter	MDI	CSII	Mann–Whitney U Test	
	Mediana (Q1–Q3)		U	*p*-Value
BMI SDS	0.65 (−0.34–1.19)	0.77 (0.22–1.21)	1138	0.4
Height, cm	157.45 (139.12–165.73)	162.9 (149–169.38)	990	0.06
Height SDS	0.65 (−0.39–1.17)	0.7 (−0.03–1.14)	1200	0.68
Tanner stage	2 (2–4)	4 (2–5)	979.5	0.049
Fasting plasma glucose, mg/dL	177.1 (157.38–194.85)	133.45 (119.32–144.45)	2323.5	<0.001
HbA1c, %	7.6 (6.88–8.31)	7.3 (6.6–8.4)	1369.5	0.4
Creatinine, mg/dL	0.56 (0.46–0.6)	0.7 (0.57–0.74)	753	<0.001
eGFR, mL/min/1.73 m^2^	118 (105.5–140)	115.5 (98.16–141.25)	1327	0.65
AST, U/L	21 (18–28.25)	21.5 (17–25.75)	1296	0.8
ALT, U/L	19 (16–23.25)	18 (15.25–20.75)	1387.5	0.38
Total cholesterol, mg/dL	167 (148–198)	162 (136.5–184.75)	1393.5	0.36
Triglycerides, mg/dL	65 (47.75–92)	59.5 (53–68.75)	1344	0.57
HDL cholesterol, mg/dL	63.9 (54–69.25)	58.2 (51–67)	1469	0.09
LDL cholesterol, mg/dL	89 (74.5–106)	89 (76.5–107.25)	1224.5	0.81

Mann–Whitney U test; MDI, multiple daily injections; CSII, continuous subcutaneous insulin infusion; BMI, body mass index; SDS, standard deviation; HbA1c, glycated hemoglobin; eGFR, estimated glomerular filtration rate (Scwartz method); ALT, alanine aminotransferase; AST, aspartate aminotransferase, HDL, high-density lipoprotein; LDL, low-density lipoprotein.

**Table 5:** Comparison of continuous glucose monitoring parameters according to pubertal stage in children treated with CSII (*n* = 42).

Parameter	Tanner I	Tanner II–IV	Tanner V	Kruskal–Wallis Test.	
				H	*p*-Value
HbA1c (%)	6.9 (6.57–7.58)	7.4 (6.8–8.2)	7.8 (6.95–8.45)	1.21	0.54
Time in Range (%)	67.5 (60.75–73.75)	70 (63–78)	76 (67–81)	5.91	0.05
Hyperglycemia 180–250 mg/dL (%)	25 (23–29.25)	20 (16–28)	14 (12–23)	6.7	0.03
Hyperglycemia > 250 mg/dL (%)	3 (2–9.25)	4 (1–10)	2 (0–9.5)	1.79	0.4
Hypoglycemia 54–70 mg/dL (%)	0 (0–1)	1 (0–2)	1 (0–3)	1.94	0.37
Hypoglycemia < 54 mg/dL (%)	2.5 (0.75–6)	1 (0–4)	1 (0.50–3.5)	0.33	0.84
BMI SDS	0.59 (0.16–1.26)	0.76 (0.12–1.09)	0.73 (−0.11–1)	1.43	0.48
Height SDS	0.85 (0.5–1.1)	0.52 (−0.09–0.92)	0.7 (−0.36–1.26)	1.07	0.58

Kruskal–Wallis test; CSII, continuous subcutaneous insulin infusion; HbA1c, glycated hemoglobin; BMI, body mass index; SDS, standard deviation.

**Table 6:** Correlation analysis for BMI SDS and Height SDS in all subjects (*n* = 177).

Parameter	BMI SDS Spearman’s ρ	*p*-Value	Height SDS Spearman’s ρ	*p*-Value
Age	−0.01	0.872	−0.13	0.07
Tanner stage	0.02	0.769	−0.13	0.07
Fasting plasma glucose	−0.06	0.379	−0.02	0.77
Creatinine	0.07	0.357	−0.04	0.54
eGFR	−0.08	0.266	0.19	0.01
AST	−0.13	0.068	0.01	0.81
ALT	0.1	0.17	0.02	0.7

Spearman correlations; BMI, body mass index; SDS, standard deviation; eGFR, estimated glomerular filtration rate (Scwartz method); ALT, alanine aminotransferase; AST, aspartate aminotransferase.

**Table 7:** Correlation analysis for BMI SDS and Height SDS in all diabetic subjects (*n* = 102).

Parameter	BMI SDS Spearman’s ρ	*p*-Value	Height SDS Spearman’s ρ	*p*-Value
Age	−0.07	0.46	−0.09	0.35
Duration of diabetes	0.09	0.37	−0.18	0.07
Tanner stage	−0.03	0.74	−0.09	0.34
HbA1c	−0.1	0.28	−0.13	0.16
Fasting plasma glucose	−0.14	0.14	−0.13	0.18
Creatinine	0.01	0.89	−0.13	0.17
eGFR	−0.16	0.09	0.19	0.05
Total cholesterol	0.1	0.3	−0.03	0.74
LDL cholesterol	0.07	0.45	−0.04	0.66
HDL cholesterol	−0.01	0.9	0.1	0.31
Triglycerides	0.05	0.56	−0.07	0.45
AST	−0.04	0.64	0.003	0.97
ALT	−0.06	0.52	−0.13	0.16

Spearman correlations; BMI, body mass index; SDS, standard deviation; HbA1c, glycated hemoglobin; eGFR, estimated glomerular filtration rate (Scwartz method); LDL, low-density lipoprotein; HDL, high-density lipoprotein; ALT, alanine aminotransferase; AST, aspartate aminotransferase.

**Table 8:** Correlation analysis for BMI SDS and Height SDS in CSII treated diabetic subjects (*n* = 42).

Parameter	BMI SDS Spearman’s ρ	*p*-Value	Height SDS Spearman’s ρ	*p*-Value
HbA1c (%)	−0.26	0.09	−0.13	0.38
Time in Range (%)	0.21	0.18	0.18	0.23
Hypoglycemia 40–54 mg/dL (%)	0.08	0.59	0.08	0.58
Hypoglycemia 54–70 mg/dL (%)	0.34	0.02	−0.04	0.76
Hyperglycemia 180–250 mg/dL (%)	−0.18	0.23	−0.17	0.26
Hyperglycemia 250–400 mg/dL (%)	−0.2	0.18	−0.22	0.14
Coefficient of variation (%)	−0.13	0.38	−0.07	0.66

Spearman correlations; BMI, body mass index; SDS, standard deviation; HbA1c, glycated hemoglobin.

**Table 9:** Multiple linear regression analysis for factors associated with BMI SDS (*n* = 177).

Predictor	B	SE	Standardized β	95% CI	*p*-Value	VIF
Age	0.01	0.06	0.028	−0.12 to 0.14	0.87	5.77
Sex	0.01	0.22	0.006	−0.42 to 0.46	0.93	1.10
Residence	−0.54	0.21	−0.190	−0.97 to −0.11	0.01	1.03
Tanner stage	0.01	0.16	0.012	−0.31 to 0.32	0.95	6.07
Fasting plasma glucose	−0.002	0.002	−0.082	−0.007 to 0.002	0.30	1.14
eGFR	−0.005	0.004	−0.094	−0.01 to 0.003	0.24	1.14
AST	0.0003	0.003	0.013	−0.006 to 0.006	0.92	3.59
ALT	−0.0004	0.004	−0.012	−0.009 to 0.008	0.93	3.51

Multiple linear regression; BMI, body mass index; SDS, standard deviation; eGFR, estimated glomerular filtration rate (Scwartz method); ALT, alanine aminotransferase; AST, aspartate aminotransferase; B, unstandardized regression coefficient; SE, standard error; β, standardized regression coefficient; CI, confidence interval; VIF, variance inflation factor.

**Table 10:** Multiple linear regression analysis for factors associated with height SDS (*n* = 177).

Predictor	B	SE	Standardized β	95% CI	*p*-Value	VIF
Age	−0.02	0.05	−0.07	−0.12 to 0.08	0.69	5.77
Sex	−0.13	0.17	−0.05	−0.48 to 0.22	0.46	1.10
Residence	0.35	0.17	0.15	0.003 to 0.69	0.04	1.07
BMI SDS	0.15	0.06	0.18	0.02 to 0.27	0.01	1.06
Tanner stage	−0.03	0.12	−0.04	−0.28 to 0.22	0.81	6.07
Fasting plasma glucose	0.0003	0.002	0.01	−0.003 to 0.004	0.87	1.14
eGFR	0.005	0.003	0.12	−0.001 to 0.012	0.12	1.15
AST	−0.001	0.002	−0.1	−0.007 to 0.003	0.46	3.59
ALT	0.002	0.003	0.08	−0.005 to 0.009	0.55	3.51

Multiple linear regression; BMI, body mass index; SDS, standard deviation; eGFR, estimated glomerular filtration rate (Scwartz method); ALT, alanine aminotransferase; AST, aspartate aminotransferase; B, unstandardized regression coefficient; SE, standard error; β, standardized regression coefficient; CI, confidence interval; VIF, variance inflation factor.

**Table 11:** Multiple linear regression analysis evaluating metabolic and treatment-related factors associated with height SDS in children with T1DM (*n* = 102).

Predictor	B	SE	Standardized β	95% CI	*p*-Value	VIF
Fasting plasma glucose	−0.003	0.007	−0.12	−0.01 to 0.01	0.63	6.03
HbA1c	0.01	0.15	0.01	−0.29 to 0.31	0.94	3.38
eGFR	0.004	0.004	0.12	−0.004 to 0.01	0.29	1.16
ALT	0.001	0.003	0.04	−0.005 to 0.007	0.71	1.61
LDL-c	−0.001	0.004	−0.05	−0.01 to 0.007	0.68	1.38
HDL-c	0.003	0.008	0.06	−0.01 to 0.02	0.65	1.36
Triglycerides	−0.002	0.002	−0.12	−0.007 to 0.003	0.39	1.85
CSII versus MDI	−0.09	0.38	−0.04	−0.86 to 0.66	0.8	3.24

Dependent variable: height SDS. MDI was coded as 0 and CSII as 1. Multiple linear regression; SDS, standard deviation; HbA1c, glycated hemoglobin; eGFR, estimated glomerular filtration rate (Scwartz method); ALT, alanine aminotransferase; LDL, low-density lipoprotein; HDL, high-density lipoprotein; CSII, continuous subcutaneous insulin infusion; MDI, multiple daily injection; B, unstandardized regression coefficient; SE, standard error; β, standardized regression coefficient; CI, confidence interval; VIF, variance inflation factor.

**Table 12:** Multiple linear regression analysis evaluating the association between glycemic control and height SDS in subjects with T1DM (*n* = 102).

Predictor	B	SE	Standardized β	95% CI	*p*	VIF
HbA1c (%)	−0.07	0.09	−0.08	−0.25 to 0.1	0.42	1.2
Age (years)	0.02	0.03	0.08	−0.04 to 0.08	0.50	1.43
Sex (female vs. male)	0.14	0.2	0.07	−0.26 to 0.55	0.49	1.08
Diabetes duration (years)	−0.05	0.03	−0.23	−0.11 to 0.008	0.08	1.54
CSII versus MDI	−0.21	0.25	−0.09	−0.72 to 0.3	0.41	1.3

Dependent variable: height SDS. MDI was coded as 0 and CSII as 1. Multiple linear regression; SDS, standard deviation; T1DM, type 1 diabetes mellitus; HbA1c, glycated hemoglobin; CSII, continuous subcutaneous insulin infusion; MDI, multiple daily injection; B, unstandardized regression coefficient; SE, standard error; β, standardized regression coefficient; CI, confidence interval; VIF, variance inflation factor.

## Data Availability

Only the corresponding author may provide access to the data reported in this study. Due to privacy limitations, the data are not publicly accessible.

## Abbreviations

ALT	Alanine aminotransferase
AST	Aspartate aminotransferase
BMI	Body mass index
BMI SDS	Body mass index standard deviation score
CGM	Continuous glucose monitoring
CSII	Continuous subcutaneous insulin infusion
CV	Coefficient of variation
eGFR	Estimated glomerular filtration rate
GH	Growth hormone
HbA1c	Glycated hemoglobin
HDL	High-density lipoprotein
IGF-1	Insulin-like growth factor 1
LDL	Low-density lipoprotein
MDI	Multiple daily injections
SDS	Standard deviation score
T1DM	Type 1 diabetes mellitus
Tanner stage	Tanner stage of pubertal development
TIR	Time in range
